# Absence of RIP140 Reveals a Pathway Regulating glut4-Dependent Glucose Uptake in Oxidative Skeletal Muscle through UCP1-Mediated Activation of AMPK

**DOI:** 10.1371/journal.pone.0032520

**Published:** 2012-02-28

**Authors:** Asmaà Fritah, Jennifer H. Steel, Nadeene Parker, Evanthia Nikolopoulou, Mark Christian, David Carling, Malcolm G. Parker

**Affiliations:** 1 Institute of Reproductive and Developmental Biology, Imperial College, London, United Kingdom; 2 Department of Cell and Developmental Biology, University College London, London, United Kingdom; 3 Medical Research Council's Clinical Sciences Centre, Faculty of Medicine, Imperial College, London, United Kingdom; Institute of Genetics and Molecular and Cellular Biology, France

## Abstract

Skeletal muscle constitutes the major site of glucose uptake leading to increased removal of glucose from the circulation in response to insulin. Type 2 diabetes and obesity are often associated with insulin resistance that can be counteracted by exercise or the use of drugs increasing the relative proportion of oxidative fibers. RIP140 is a transcriptional coregulator with a central role in metabolic tissues and we tested the effect of modulating its level of expression on muscle glucose and lipid metabolism in two mice models. Here, we show that although RIP140 protein is expressed at the same level in both oxidative and glycolytic muscles, it inhibits both fatty acid and glucose utilization in a fiber-type dependent manner. In RIP140-null mice, fatty acid utilization increases in the extensor digitorum longus and this is associated with elevated expression of genes implicated in fatty acid binding and transport. In the RIP140-null soleus, depletion of RIP140 leads to increased GLUT4 trafficking and glucose uptake with no change in Akt activity. AMPK phosphorylation/activity is inhibited in the soleus of RIP140 transgenic mice and increased in RIP140-null soleus. This is associated with increased UCP1 expression and mitochondrial uncoupling revealing the existence of a signaling pathway controlling insulin-independent glucose uptake in the soleus of RIP140-null mice. In conclusion, our findings reinforce the participation of RIP140 in the maintenance of energy homeostasis by acting as an inhibitor of energy production and particularly point to RIP140 as a promising therapeutic target in the treatment of insulin resistance.

## Introduction

Skeletal muscle constitutes the major site of glucose uptake leading to increased removal of glucose from the circulation in response to insulin. Insulin resistance is a key feature of type 2 diabetes and obesity where it is often associated with accumulation of intramyocellular lipids and decreased oxidative capacities in skeletal muscle. Skeletal muscles required for sustained contractile activity such as the soleus contain mainly “slow twitch” oxidative fibers, rich in mitochondria, while those involved in rapid, shorter bursts of activity such as the gastrocnemius and extensor digitorum longus (EDL) contain more “fast twitch” fibers rich in glycolytic enzymes for anaerobic metabolism [1]. Slow fibers tend to express type I myosin heavy chains (MyHC) while fast fibers express IIa, IIb and IIx isoforms [1]–[3]. Fiber type composition can be modulated by different factors such as exercise, aging, and hormonal changes [4], [5]. Endurance training leads to an increase in the proportion of type I fibers while resistance training promotes increased type II fibers. Although it has been shown that increasing the proportion of the most oxidative fibers helps in counteracting diet-induced obesity [6], increasing type II/glycolytic fibers promotes beneficial effects on obesity and associated metabolic disorders [7].

In recent years, AMP-activated protein kinase (AMPK) has emerged as a critical regulator of skeletal muscle oxidative function [8]. The energy sensing capabilities of AMPK are attributed to its ability to detect and react to fluctuations in the AMP/ATP ratio that take place during rest and exercise. AMPK is a heterotrimer comprised of catalytic α- and regulatory β- and γ-subunits [9]. AMPK is activated by phosphorylation of threonine 172 (T172) within the T-loop of the α-subunit catalyzed by either LKB1 or Ca^2+^/calmodulin dependent protein kinase kinase (CaMKK) β [10]–[12]. Recent studies have shown that the major mechanism for activation of AMPK in response to ATP depletion is *via* binding of AMP to the regulatory γ subunit and protection against dephosphorylation of T172 [13]. Once activated, AMPK restores cellular energy balance by regulating transcription as well as activity of enzymes implicated in the control of fatty acid and glucose utilization. It has been accepted for long time that AMPK acts on fatty acid transport into the mitochondria *via* phosphorylation and inhibition of acetyl-CoA carboxylase (ACC) which converts acetyl-CoA to malonyl-CoA, an inhibitor of carnitine palmitoyltransferase-1 (CPT-1) [14] thus leading to subsequent oxidation of fatty acids. Interestingly, there is growing body of evidence that AMPK and/or ACC phosphorylation does not systematically correlate with increased fatty acid oxidation suggesting the existence of additional kinases and/or signaling pathways implicated in this process [15]. In addition, AMPK acts on glucose utilization by increasing the expression of glut4 and its translocation to the plasma membrane leading to increased glucose uptake in skeletal muscle, independently of insulin [16], [17]. This mode of action is consistent with recent evidence indicating that AMPK plays a role in the therapeutic benefits of exercise [14], [18] as well as metformin [19], [20] in the treatment of type 2 diabetes and associated metabolic disorders.

Recent evidence indicates that Receptor-interacting protein 140 (RIP140) is a multi-functional coregulator with a central role in metabolic tissues [21]–[25]. It binds and represses a number of nuclear receptors including the peroxisome proliferator activated receptors (PPARα, PPARβ/δ, and PPARγ) [26], thyroid hormone receptors (TRα, and TRβ) [27] and estrogen-related receptors (ERRα, and ERRβ) [28] particularly important in regulating metabolic gene expression in adipose tissue, muscle and liver [29]. The metabolic properties of skeletal muscle can be regulated by nuclear receptors with coactivators playing a key role [6], [30]–[32]. Similarly, transcriptional repression by nuclear receptors is now emerging as a key contributor to muscle physiology [33]. There is substantial evidence to indicate that RIP140 functions as a corepressor in skeletal muscle to control fiber type switching and metabolism [25], [34], [35].

Previously, we studied the effect of RIP140 in skeletal muscle, through loss or gain of function using RIP140-null and transgenic mice [25]. The design of the study was based on the endogenous level of RIP140 mRNAs, higher in glycolytic compared to oxidative muscles, and led to the main conclusion that RIP140 acts as a transcriptional corepressor that inhibits oxidative metabolism [25]. In addition to supporting this conclusion, the data presented in this study show that absence of RIP140 reveals an unexpected pathway regulating glucose uptake in the soleus.

## Results

### RIP140 participates in the glycolytic phenotype of the EDL by inhibiting fatty acid utilization

**Figure 1:**
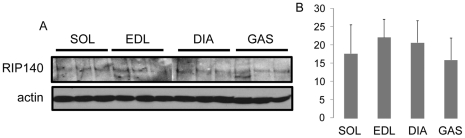
RIP140 protein level is the same in oxidative and glycolytic muscles. (A) Analysis of soleus (SOL), EDL, diaphragm (DIA), and gastrocnemius (GAS) protein extracts by western-blot for RIP140 and actin expression. (B) Quantification of the western-blot for RIP140. Data are expressed as mean ± SEM.

Previously, we have shown that RIP140 mRNA levels are higher in glycolytic muscles, EDL and gastrocnemius, compared to oxidative ones, soleus and diaphragm [25]. While further investigating RIP140 expression in the same tissues, we were surprised to observe no significant difference in RIP140 protein levels among tested skeletal muscles (Figure 1A, B). Therefore, as there is no gradient of RIP140 protein expression in skeletal muscles we were prompted to reassess RIP140 function as a negative regulator of energy metabolism in both oxidative and glycolytic muscles.

**Figure 2:**
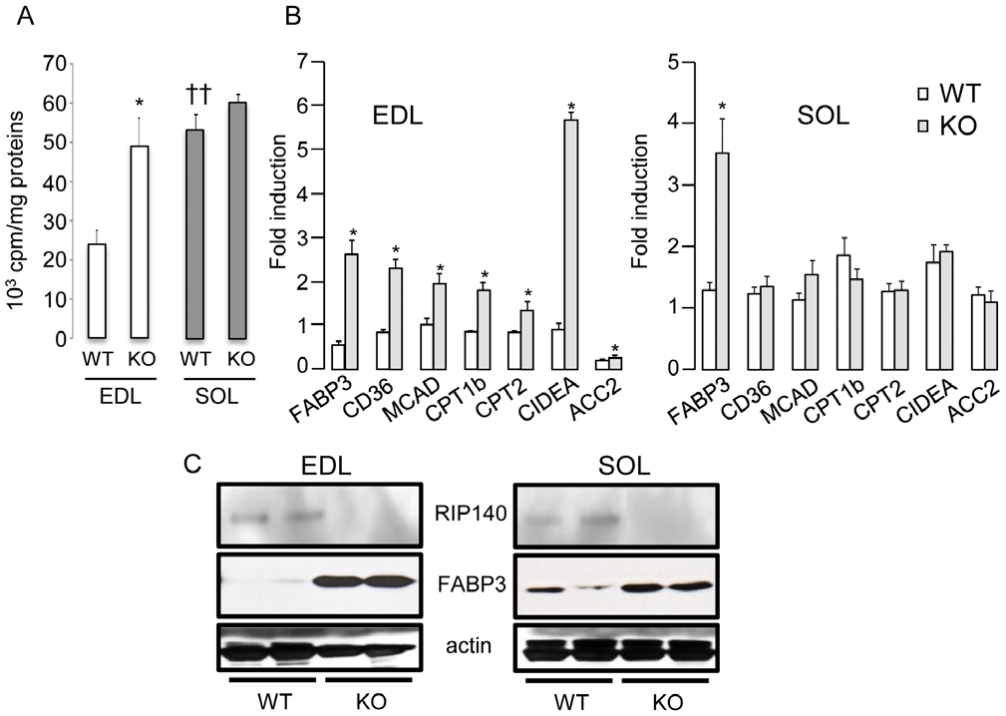
Absence of RIP140 increases fatty acid utilization in the EDL. (A) β-oxidation rate, normalized to proteins, monitored in EDL and soleus (SOL) isolated from RIP140-null (KO) and WT mice (n = 4). Data are expressed as mean ± SEM. *p<0.05, WT *vs* KO; ††p<0.01, WT soleus *vs* WT EDL. (B) Real-time RT-PCR analysis of genes implicated in fatty acid binding and transport in EDL and soleus (SOL) of RIP140-null (KO) and WT mice. Data are comparable between histograms for the same gene and expressed as mean ± SEM. *p<0.05, WT *vs* KO. (C) Analysis of RIP140, FABP3 and actin expression in RIP140-null (KO) and WT EDL and soleus (SOL) by western blot.

**Figure 3:**
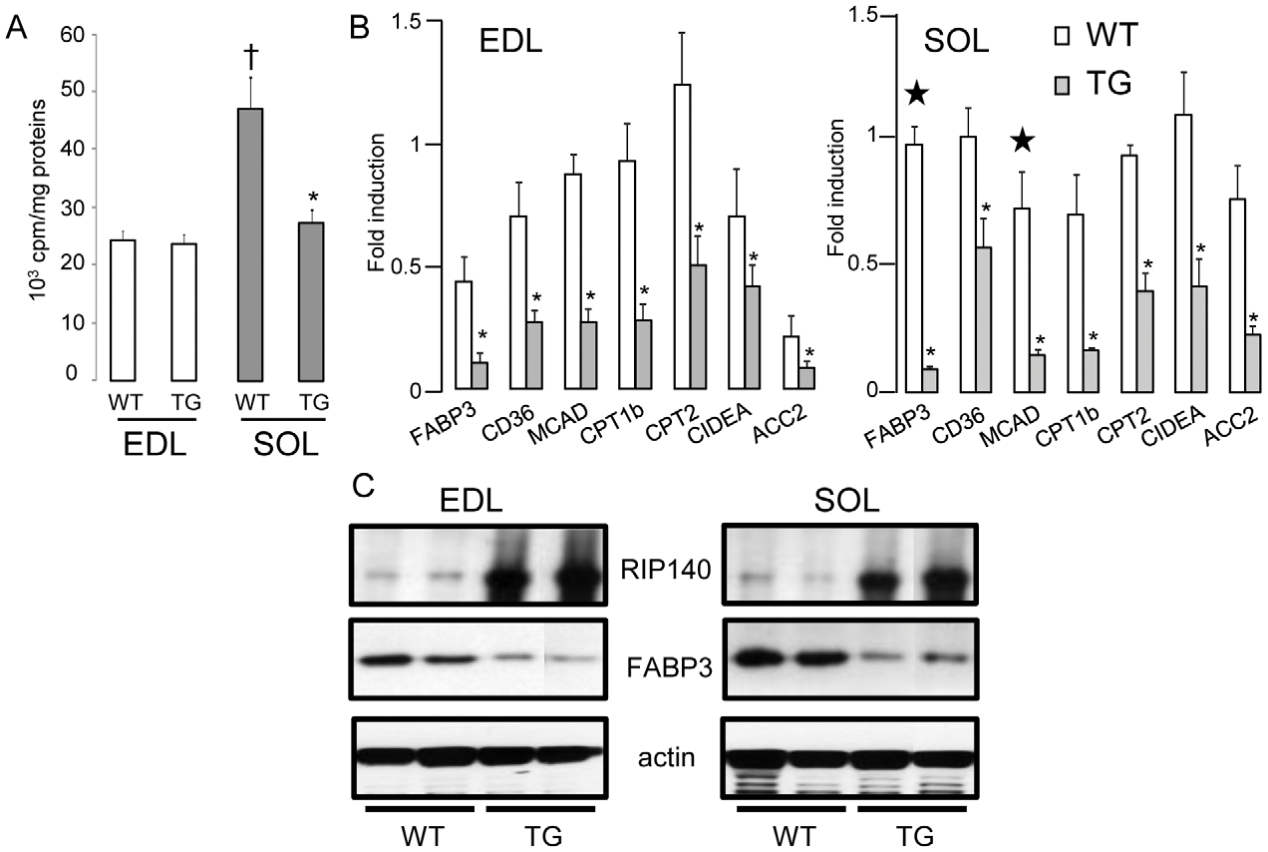
Exogenous expression of RIP140 inhibits fatty acid utilization in the soleus. (A) β-oxidation rate, normalized to proteins, monitored in EDL (n = 8) and soleus (SOL) (n = 4) isolated from RIP140 transgenic (TG) and WT mice. Data are expressed as mean ± SEM. *p<0.05, WT *vs* KO; †p<0.05, WT soleus *vs* WT EDL. (B) Real-time RT-PCR analysis of genes implicated in fatty acid binding and transport in EDL and soleus (SOL) of RIP140 transgenic (TG) and WT mice. Data are comparable between histograms for the same gene and expressed as mean ± SEM. *p<0.05, WT *vs* TG. ???Data were published already in [25]. (C) Analysis of RIP140, FABP3 and actin expression in RIP140 transgenic (TG) and WT EDL and soleus (SOL) by western blot.

We analyzed the effect of modulating RIP140 levels on the rate of β-oxidation in isolated EDL and soleus. Absence of RIP140 induced increased β-oxidation in the glycolytic-rich EDL to a level similar to that observed in the oxidative-rich soleus (Figure 2A). In WT muscles, β-oxidation was two- to three-fold lower in the EDL compared to the soleus (Figure 2A). Analysis of mRNA levels of key metabolic genes implicated in fatty acid handling showed that FABP3, CD36, MCAD, CPT1b, CPT2, CIDEA and ACC2 were increased in the absence of RIP140 in the EDL while unaffected in the soleus, except for FABP3 (Figure 2B). Protein levels for RIP140 and FABP3, a key target implicated in fatty acid transport, were analyzed and confirmed the changes observed at the mRNA level (Figure 2C). In contrast, absence of RIP140 did not affect β-oxidation in the soleus suggesting that RIP140 does not suppress fatty acid utilization in this oxidative muscle (Figure 2A). To further characterize the role of RIP140 in metabolic regulation, we monitored the effect of exogenous expression of RIP140 in muscles from a RIP140 transgenic mouse model. Exogenous expression of RIP140 decreased the rate of β-oxidation in the soleus to a level similar to that observed in the EDL (Figure 3A). No change in β-oxidation rate is observed in the EDL, indicating that maximal repression of fatty acid utilization is exerted in this muscle. In both muscles, exogenous RIP140 caused reduced expression of genes/proteins implicated in fatty acid transport (Figure 3B–C). These data show that RIP140 acts as a transcriptional corepressor that maintains the glycolytic properties of the EDL by inhibiting fatty acid utilization.

**Figure 4:**
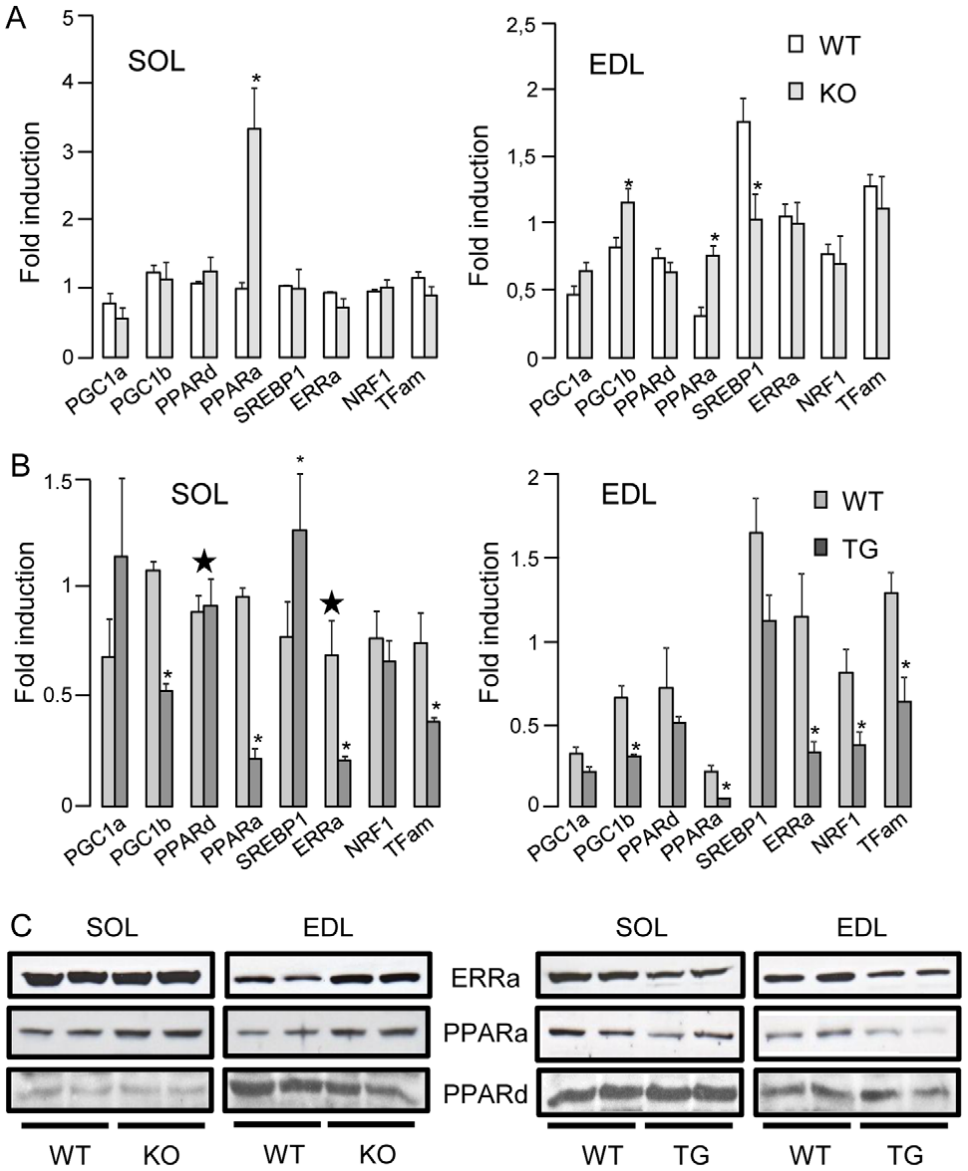
Modulation of RIP140 alters the expression of metabolic nuclear receptors and transcription factors. (A) Real-time RT-PCR analysis of metabolic nuclear receptors and transcription factors in soleus (SOL) and EDL of RIP140-null (KO) and WT mice. Data are comparable between histograms for the same gene and expressed as mean ± SEM. *p<0.05, WT *vs* KO. (B) Real-time RT-PCR analysis of metabolic nuclear receptors and transcription factors in soleus (SOL) and EDL of RIP140 transgenic (TG) and WT mice. Data are comparable between histograms for the same gene and expressed as mean ± SEM. *p<0.05, WT *vs* TG. ???Data were published already in [25]. (C) Analysis of ERRα, PPARα, and PPARβ/δ expression in RIP140-null (KO), transgenic (TG) and WT soleus (SOL) and EDL by western blot.

RIP140 has been shown to act as a transcriptional corepressor for metabolic nuclear receptors ERRs, PPARs as well as for NRF1 and TFam, two transcription factors controlling mitochondrial function and biogenesis. Analysis of mRNA expression in the soleus and EDL showed that overexpression of RIP140 reduced levels of ERRα, PPARα and TFam while absence of RIP140 upregulated PPARα mRNAs (Figure 4A–C) suggesting that RIP140 regulates the expression of these genes similarly in both muscles. In parallel, absence of RIP140 did not affect the expression of PPARβ/δ (Figure 4A, C) and NRF1 (Figure 4A) in both muscles while overexpression of RIP140 inhibited NRF1 mRNAs only in the EDL (Figure 4B). Contrary to other targets, SREBP1, implicated in fatty acid synthesis, is upregulated when RIP140 is overexpressed in the soleus while absence of RIP140 inhibits its expression in the EDL (Figure 4A, B). This is in agreement with RIP140 acting as a coactivator for LXR in regulating the expression of SREBP1 in the liver [22]. In addition, the expression of the metabolic coactivator PGC-1β is downregulated by RIP140 overexpression in both muscles while upregulated by the depletion of RIP140 only in the EDL (Figure 4A, B). The difference observed in the level of NRF1, SREBP1 and PGC-1β in the EDL and soleus suggests that RIP140 acts in a fiber type-specific manner to regulate the expression of these genes. Thus, RIP140 functions as a corepressor in both the EDL and soleus regulating the expression of a number of transcription factors and nuclear receptors but it may also function as a coactivator, for example for SREBP1 gene, thereby acting as an important regulator of metabolic gene expression.

### Depletion of RIP140 increases GLUT4 trafficking and basal glucose uptake in the soleus through activation of AMPK

**Figure 5:**
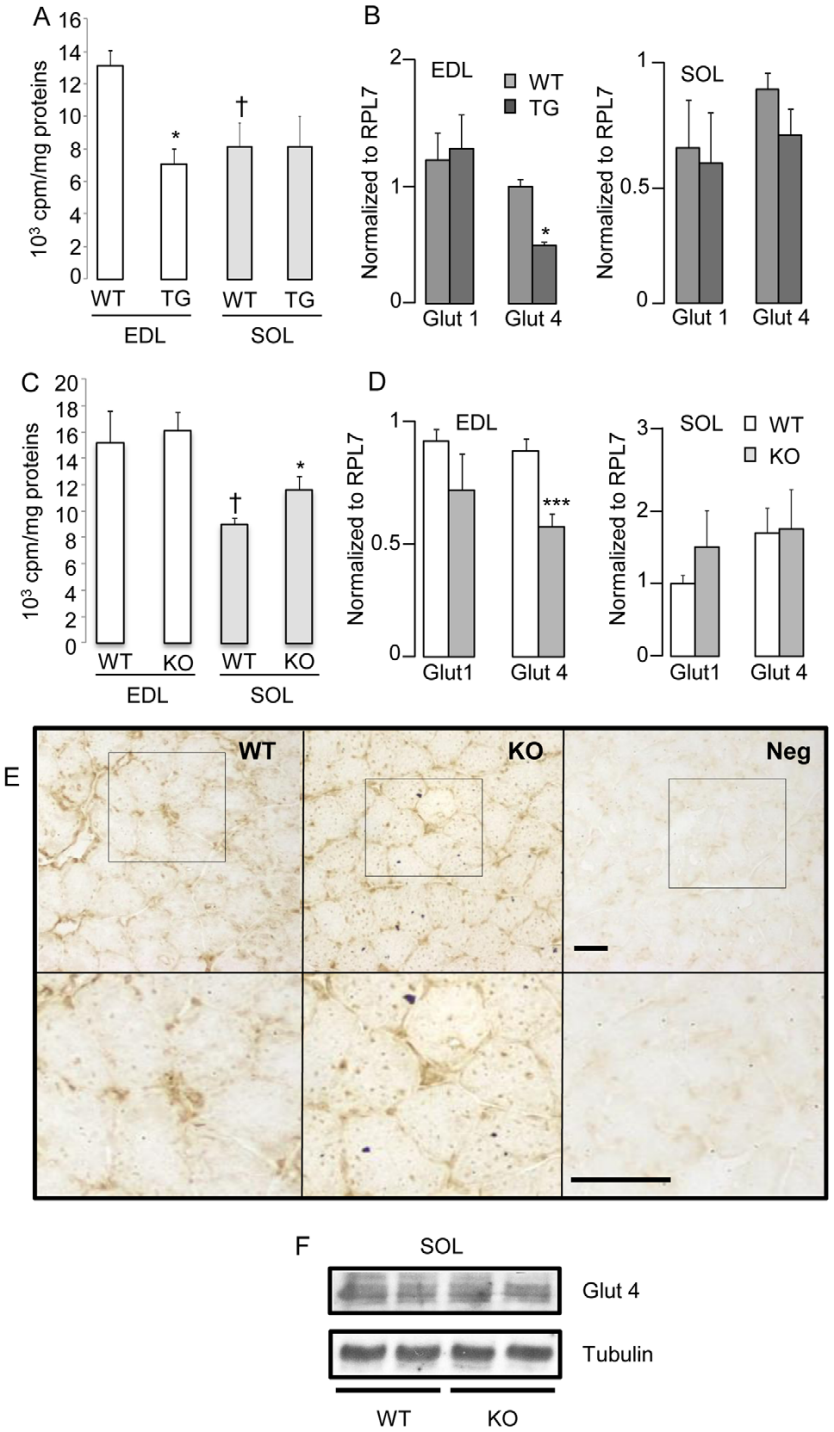
Depletion of RIP140 increases the presence of GLUT4 at the plasma membrane and basal glucose uptake in the soleus. (A) Basal glucose uptake, normalized to proteins, monitored in EDL (n = 4) and soleus (SOL) (n = 8) isolated from RIP140 transgenic (TG) and WT mice. Data are expressed as mean ± SEM. *p<0.05, WT *vs* KO; †p<0.05, WT soleus *vs* WT EDL. (B) Real-time RT-PCR analysis of genes implicated in glucose transport in EDL and soleus (SOL) of RIP140 transgenic (TG) and WT mice (n = 4). Data are expressed as mean ± SEM, *p<0.05, WT *vs* TG. (C) Basal glucose uptake, normalized to proteins, monitored in EDL and soleus (SOL) isolated from RIP140-null (KO) *versus* WT mice (n = 4–8). Data are expressed as mean ± SEM, *p<0.05, WT *vs* KO; †p<0.05, WT EDL *vs* WT soleus. (D) Real-time RT-PCR analysis of genes implicated in glucose transport in EDL and soleus (SOL) of RIP140-null (KO) and WT mice (n = 4). Data are expressed as mean ± SEM, ***p<0.001, WT *vs* KO. (E) Immunohistochemistry for GLUT4 on RIP140-null (KO) and WT soleus. Lower panels show increased magnification of upper panels (scale bar = 100 µm). (F) Analysis of GLUT4 and tubulin expression in RIP140-null (KO) and WT soleus (SOL) by western blot.

Mice devoid of RIP140 display enhanced glucose tolerance and responsiveness to insulin compared to WT littermates [24]. To define comprehensively the metabolic role of RIP140 in muscle, we investigated changes in glucose disposal by skeletal muscles at rest and analyzed the effect of RIP140 on glucose uptake. Overexpression of RIP140 resulted in decreased 2-deoxyglucose uptake in the EDL, with negligible change in the soleus (Figure 5A). In parallel, expression analysis shows that the insulin-independent glucose transporter, glut1, was not affected (Figure 5B). Interestingly, the profile of glut4 mRNAs (lower in the RIP140 transgenic EDL and unaffected in the soleus) correlates with that of glucose uptake in the same muscles overexpressing RIP140 (Figure 5A–B and Figure S1) suggesting that RIP140 controls glucose uptake through the regulation of glut4 expression.

Next, we investigated how depletion of RIP140 might affect glucose transport in RIP140-null skeletal muscles. Glucose uptake was increased in the isolated soleus of RIP140-null mice with no change in the EDL (Figure 5C) suggesting that endogenous RIP140 participates in the oxidative phenotype of the soleus by inhibiting glucose uptake. In RIP140-null isolated muscle, in contrast to RIP140 transgenic muscles, changes in glucose uptake only partially rely upon changes in glucose transporter mRNAs (Figure 5C–D). Thus, we hypothesized that GLUT4 sub-cellular localization might be affected as it is known to be subject to regulation. Increased GLUT4 immunoreactivity was detected as increased granular staining observed in the sarcoplasma and associated with the plasma membrane of myotubes in the soleus of RIP140-null mice (Figure 5E) whereas glut4 mRNA and protein expression was unaltered (Figure 5D, F). Together, these data show that RIP140 controls glucose uptake through regulation of glut4 expression and sub-cellular localization in skeletal muscle.

**Figure 6:**
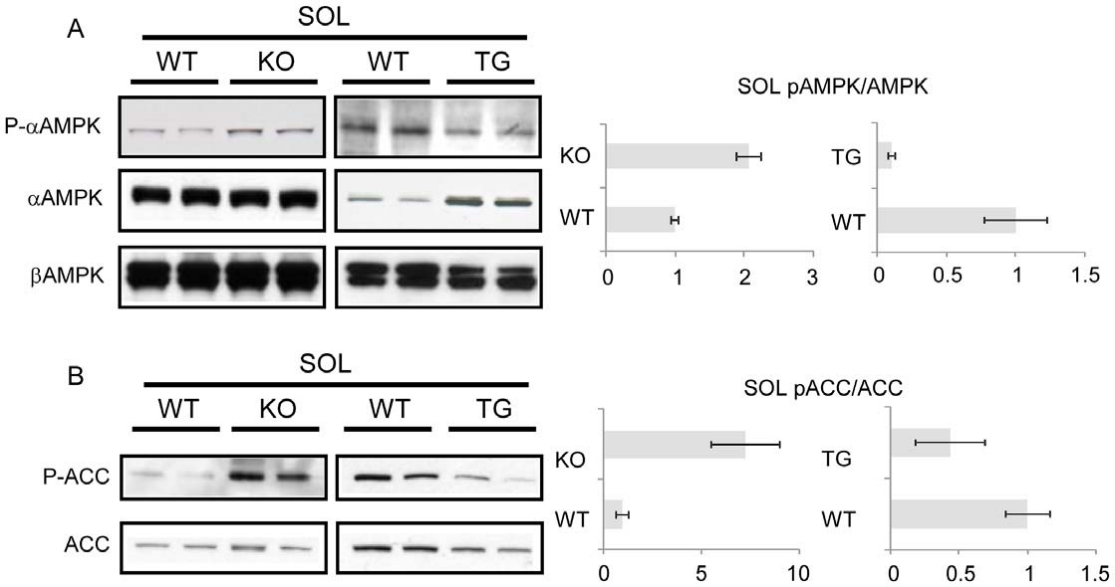
RIP140 modulates AMPK activity. (A) Analysis of phospho-αAMPK, total α and β AMPK expression on RIP140-null (KO), transgenic (TG) and WT soleus (SOL) by western-blot. Quantification of phospho-AMPK to AMPK ratio for western-blot expressed as mean ± SEM. (B) Analysis of phospho-ACC and total ACC expression on RIP140-null (KO), transgenic (TG) and WT soleus (SOL) by western-blot. Quantification of phospho-ACC to ACC ratio for western-blot expressed as mean ± SEM. Data are presented with WT set to one.

GLUT4 trafficking is controlled by two independent pathways (i) insulin, predominantly *via* the PI3K/Akt signaling pathway and (ii) AMPK. First, we investigated Akt activity (reflected by the phospho-Akt to Akt ratio) in the soleus and EDL and found it was unaffected by depletion as well as by exogenous expression of RIP140 (Figure S2A–B). This is consistent with previous data failing to show any alteration in Akt phosphorylation/activity in RIP140-null adipocytes [24]. In contrast to the Akt finding, absence of RIP140 led to increased AMPK activity as interpreted by increased phospho-AMPK to AMPK (Figure 6A) and phospho-ACC to ACC ratios (Figure 6B) in the soleus suggesting that endogenous RIP140 inhibits AMPK phosphorylation and thus activity. Consistent with this finding, exogenous RIP140 expression decreased AMPK and subsequent ACC phosphorylation in the soleus (Figure 6A–B). Interestingly, expression of exogenous RIP140 led to upregulation of total AMPK α-subunit proteins in the soleus (Figure 6A). In the EDL, absence of RIP140 led to increased AMPK phosphorylation (Figure S3) whereas in RIP140 transgenic mice, little change was observed in AMPK phosphorylation and total α-subunit proteins in the same muscle (Figure S3). We ruled out RIP140-dependent inhibition of LKB1 expression as the mechanism affecting AMPK activity as LKB1 expression was unaltered in either RIP140-null and transgenic muscles (data not shown). This suggests that another mechanism is responsible for the regulation of AMPK phosphorylation/activity by RIP140.

### Absence of RIP140 leads to expression of UCP1 in the soleus

**Figure 7:**
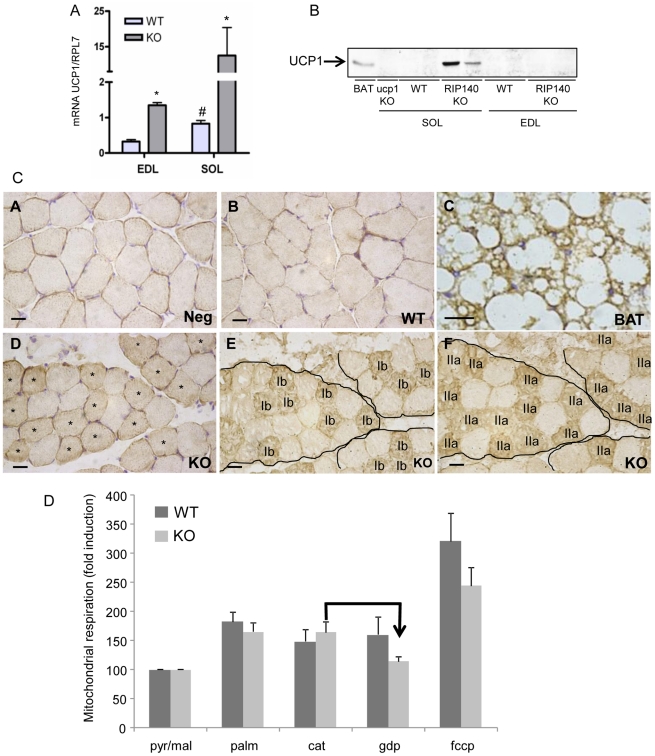
Absence of RIP140 leads to fiber-type specific expression of UCP1 in the soleus. (A) Real-time RT-PCR analysis of UCP1 mRNA level in EDL and soleus (SOL) of RIP140-null (KO) *versus* WT mice (n = 5). Data are expressed as mean ± SEM, *p<0.05, WT *vs* KO; #p<0.05, WT EDL *vs* WT soleus. (B) Analysis of UCP1 expression by western-blot on 50 µg of proteins extracted from UCP1-null soleus (ucp1 KO), RIP140-null (KO) and WT soleus, RIP140-null (KO) and WT EDL, except for brown adipose tissue (BAT, 1 µg). (C) Immunohistochemistry for UCP1 on WT and RIP140-null (KO) soleus (panels B and D, respectively) with brown adipose tissue as positive control for UCP1 (BAT, panel C) and WT soleus incubated with primary antibody omitted as negative control (Neg, panel A). Immunohistochemistry for MyHCIb (panel E) and MyHCIIa (panel F) on RIP140-null soleus. *Asterisks represent fibers positive for both UCP1 and MyHCIIa (scale bar = 100 µm). (D) Respiration of skinned myofibers isolated from RIP140-null (KO) and WT soleus was monitored after successive addition of pyruvate/malate (pyr/mal), palmitate (palm), carboxyatractylate (cat), gdp and fccp. Data are expressed as fold induction ± SEM, with respiration after addition of pyruvate/malate set to one for both RIP140-null and WT conditions.

The activity of AMPK is inhibited by elevated intracellular ATP production. Paradoxically, in RIP140-null muscles, AMPK activity is upregulated together with β-oxidation and glucose uptake, processes known to produce ATP. Thus, we postulated that a mechanism mimicking a defect in ATP production, i.e mitochondrial uncoupling, could be responsible for AMPK activation. Recently, we have shown that RIP140 acts as a transcriptional corepressor for UCP1 and that depletion of RIP140 in white adipose tissue leads to ectopic expression of UCP1 [21], [24], [36]. Although UCP1 is a specific marker of brown adipose tissue [37], we found increased UCP1 mRNA in skeletal muscles of RIP140-null soleus and EDL compared to WT (Figure 7A). When analyzed by western blot, UCP1 protein was detectable in the soleus of RIP140-null mice but not the EDL (Figure 7B). In addition, we checked for UCP1 proteins by immunohistochemistry. In the soleus, a subset of RIP140-null myofibers was stained positive for UCP1 when compared to the negative control or WT (Figure 7C panels A, B, D and Figure S4). The UCP1 staining of RIP140-null myofibers was much lower than detected in brown adipose tissue, used as a positive control (Figure 7C panels C, D). Analysis of MyHC profile showed that fibers positive for UCP1 were also positive for MyHCIIa (Figure 7C panels D, F). Expression of other mitochondrial uncoupling proteins such as ANT1, ANT2, UCP2 and UCP3 were unaffected by the absence of RIP140 in the soleus (Figure S5). In order to get functional insight about potential uncoupling, we have tested the respiration capacities of RIP140-null myofibers isolated from the soleus. Respiration of WT and RIP140-null permeabilized myofibers, isolated from the soleus, was initiated with pyruvate and uncoupled respiration was induced by addition of palmitate with no difference observed (Figure 7D). Addition of CAT (carboxyatractylate, a specific inhibitor of ANT proteins that might be responsible for uncoupling respiration in our conditions) slightly decreases WT myofibers respiration with no effect on RIP140-null one (Figure 7D). However, in the presence of GDP (a specific inhibitor of UCP1 function), we observed a decrease, although not significant, of palmitate-induced respiration of RIP140-null myofibers, but not of WT, suggesting that there might be increased UCP1 uncoupling activity in RIP140-null permeabilized myofibers (Figure 7D). Altogether these data show that absence of RIP140 leads to a fiber type-dependent increase in UCP1 expression associated with potential increased uncoupling activity.

### RIP140 inhibits UCP1 promoter activity in C2C12 cells

**Figure 8:**
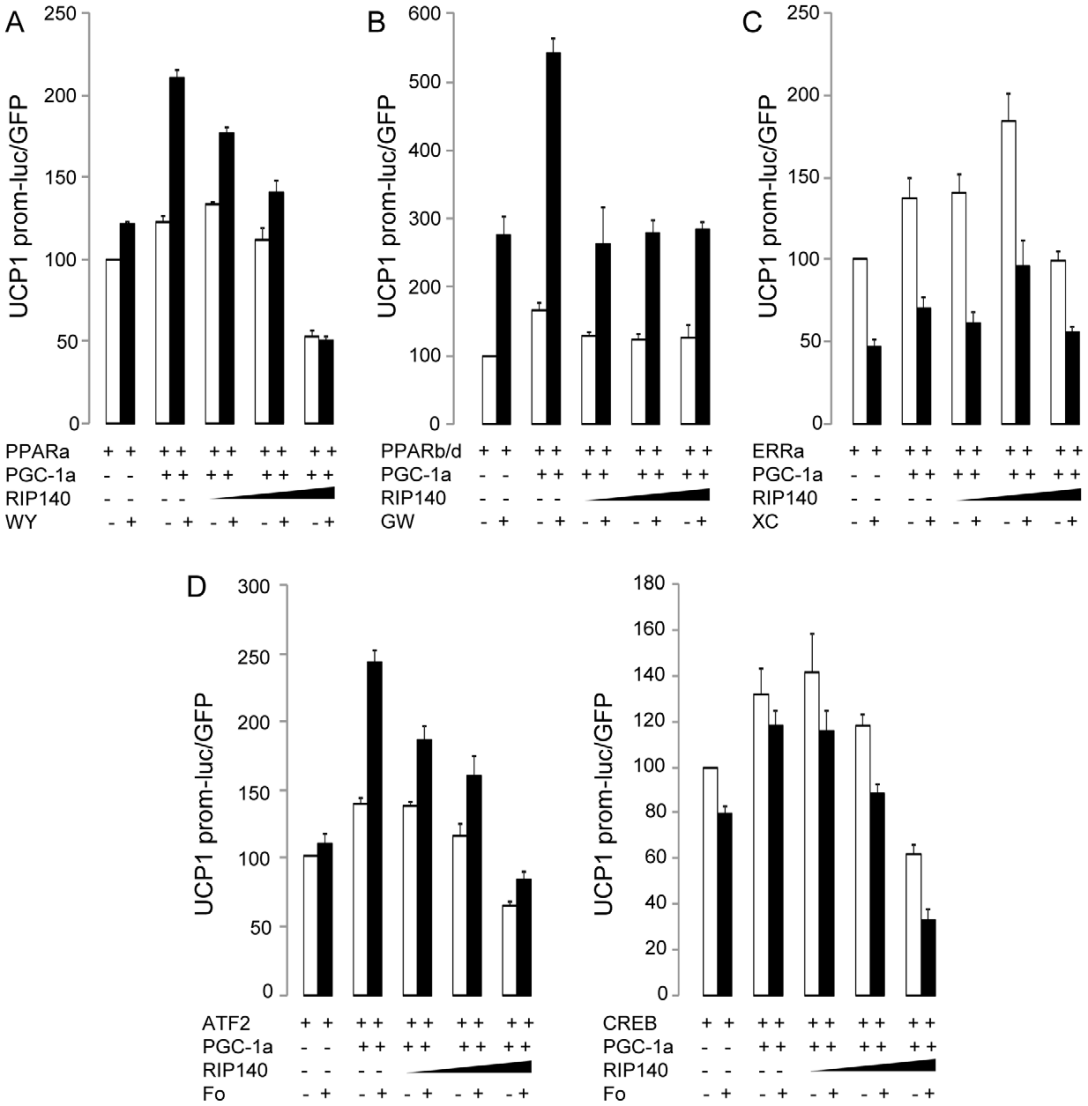
UCP1 promoter activity is induced by PGC-1α and inhibited by RIP140 in C2C12. Cells were transfected with UCP1(4 kb)-luciferase reporter and vectors expressing (A) PPARα, (B) PPARβ/δ, (C) ERRα, (D) CREB or ATF2 with or without a vector expressing PGC-1α, and increasing amount of RIP140 (0,01∶1; 0,1∶1 and 1∶1 relative to PGC-1α), and treated with (A) 100 µM WY14643 (WY), (B) 10 µM GW501516 (GW), (C) 10 µM XCT790 (XC), (D) 10 µM Forskolin (Fo) or vehicle (-) as indicated. Data are expressed as mean ± SEM.

Recently, we demonstrated that RIP140 regulates UCP1 expression through histone and DNA methylation in adipocytes [36]. Although we detected hypomethylation of the UCP1 promoter in the soleus, there was no change in the methylation status of the proximal promoter in RIP140-null compared to WT soleus in bisulfite sequencing experiments (Figure S6). To determine if UCP1 is regulated in muscle cells by PGC-1α and RIP140 in the same way as in adipocytes, a UCP1 promoter reporter construct was transfected into C2C12 cells together with PPARα, PPARβ/δ or ERRα, well-known activators of UCP1 expression in adipocytes. When cells were treated with agonists for PPARα or PPARβ/δ, WY14643 and GW501516 respectively, UCP1 promoter activity was induced modestly (Figure S7A, B). Cotransfection of PPARα or PPARβ/δ expressing vectors increased further basal and ligand-induced promoter activity (Figure S7A, B). In order to mimic an oxidative muscle environment, RIP140 protein level was kept constant and cells were transfected with PGC-1α. Addition of PGC-1α potentiated UCP1 promoter activity in the presence of PPARα and PPARβ/δ (Figure 8A, B) and increasing amount of RIP140 lead to a dose-dependent inhibition of UCP1 promoter activity in the presence of PPARα (Figure 8A). In the presence of PPARβ/δ, the minimal dose of RIP140 (0.01∶1) relative to PGC-1α inhibited the activity of the promoter (Figure 8B). UCP1 promoter activity was increased by transfection of ERRα and antagonized by the addition of the inverse agonist XCT790 (Figure S7C and 8C). Addition of PGC-1α upregulated ERRα-induced promoter activity and partially alleviated the downregulation observed in the presence of XCT790 (Figure 8C). In the presence of ERRα, the maximum dose of RIP140 (1∶1) relative to PGC-1α inhibited the activity of UCP1 promoter. Although the three nuclear receptors are capable of regulating UCP1 expression, PPARβ/δ transcriptional activity stimulated by PGC-1α seems to be the most sensitive to the inhibition exerted by RIP140 in muscle cells.

In brown adipose tissue, UCP1 expression is induced by cold through activation of the β-adrenergic signaling pathway resulting in the activation of CREB and ATF2 transcription factors. Treatment of the cells with forskolin, an activator of cAMP production and subsequent activation of CREB, led to a two-fold increase in UCP1 promoter activity (Figure S7D). When CREB or ATF2 were expressed, UCP1 promoter activity was upregulated by up to 13 fold, compared to three-fold with PPARβ/δ (Figure S7B, D). Although addition of forskolin had no effect on CREB-induced promoter activity, increased expression of PGC-1α potentiated and RIP140 inhibited, in a dose-dependent manner, the activity of UCP1 promoter in the presence of both CREB and ATF2 transcription factors (Figure 8D). Altogether these data show that expression of UCP1 in muscle cells is regulated by the same factors as in adipocytes suggesting that cold might be capable of inducing UCP1 expression in muscle cells and that RIP140 acts as a transcriptional corepressor for all of them.

## Discussion

**Figure 9:**
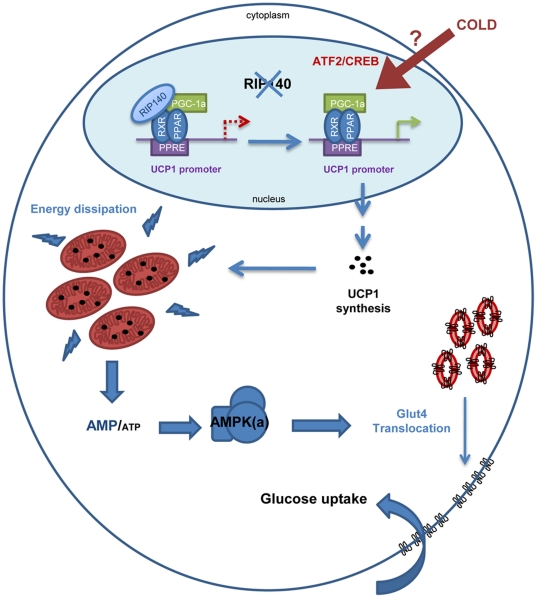
Schematic representation of the different steps leading to glucose uptake due to depletion of RIP140 in oxidative muscle. Absence of RIP140 leads to expression of UCP1 and mitochondrial uncoupling. Then, alteration of the AMP to ATP ratio in favor of elevated AMP activates AMPK which in turn stimulates GLUT4 translocation to the plasma membrane enabling the entry of glucose from blood circulation.

In this study, we show that RIP140 inhibits glucose uptake in the soleus and participates in the maintenance of its oxidative properties. Importantly, we show that depletion of RIP140 unmasks a signaling pathway upregulating insulin-independent glucose uptake in oxidative muscle through increased expression of UCP1, activation of AMPK, and, increased trafficking of GLUT4 (Figure 9).

The gene encoding RIP140 contains the full protein coding sequence within a single exon, designated exon 5, but has multiple promoters and 5′ non-coding exons that are subject to tissue- and cell-specific alternative splicing [38]. Expression analysis showed a difference in RIP140 mRNAs between skeletal muscles, higher in glycolytic muscles, such as the gastrocnemius and the EDL, and lower in oxidative-rich muscles, like the soleus and diaphragm [25]. Quantitative RT-PCR experiments were performed using a set of primer specifically targeting exon 5 enabling the detection of all RIP140 mRNA species present in skeletal muscles and potentially subject to translation [25]. Because the difference observed for RIP140 mRNAs between skeletal muscles does not seem to be translated into proteins, this suggests that post-transcriptional and/or post-translational modifications may account for this discrepancy. The existence of microRNAs targeting RIP140, such as miR346 discovered in the brain, that increases the stability of RIP140 proteins [39], might explain the discrepancy observed between skeletal muscles. Interestingly, the expression of such microRNAs should be subject to fiber type-dependent regulation to satisfy the hypothesis. Alternatively, it has been demonstrated that RIP140 is subject to ubiquitination and degradation via the ubiquitin/proteasome pathway [40] enabling the control of RIP140 activity via the regulation of its half-life. Fiber type-dependent ubiquitination/degradation has been implicated in the regulation of other transcriptional corepressors, class II HDACs, in skeletal muscles [41] and the effect on RIP140 has yet to be tested. Although total RIP140 protein levels are similar in type I- and type II-containing muscles, there are still questions concerning the distribution of RIP140 proteins among fibers and this is dependent on the availability of antibodies of high quality for immunohistochemistry/immunofluorescence experiments. Altogether, these are pertinent questions that are under investigation.

Most of the genes affected by the absence of RIP140 are upregulated which is consistent with RIP140 acting mainly as a transcriptional corepressor. In addition, most of the genes repressed by RIP140 are activated by PGC-1α, the transcriptional coactivator being expressed at higher level in oxidative muscles compared to glycolytic ones [32]. Recently, we have shown that RIP140 interacts directly with PGC-1α and suppresses its activity [42] strengthening the mutually antagonistic functions of the two coregulators. This raises the possibility that direct antagonism of PGC-1α by RIP140 may provide the basis of a fiber type-dependent mechanism for tight regulation of metabolic gene expression. In the soleus, removing the corepressor RIP140 does not alter the expression of target genes suggesting that the balance of metabolic cofactors is in favor of the pro-oxidative ones such as PGC-1α. On the other hand, metabolic gene expression and subsequent β-oxidation is downregulated in RIP140 transgenic soleus suggesting that increased expression of RIP140 enables the titration of PGC-1α, or other pro-oxidative factors, sequestering them away from target gene promoters. In the EDL, removal of RIP140 induces the upregulation of metabolic genes and subsequent β-oxidation without altering the uptake of glucose. This is in line with endogenous RIP140 acting at the transcriptional level to maintain the glycolytic properties of the EDL through inhibition of β-oxidation. However, increasing RIP140 level leads to the decrease of genes involved in the regulation of fatty acid uptake and transport in the transgenic EDL, without affecting the rate of β-oxidation. This suggests that other regulators are rate-limiting and might be acting to maintain a basal state of β-oxidation in this glycolytic muscle that is resistant to the action of increased RIP140. This threshold is a brake that might rely on the control of enzymatic activities directly involved in fatty acid utilization. This is compatible with the existence of a balance between RIP140 and PGC-1α that ensures the fine tuning of skeletal muscle metabolism in response to environmental changes. Interestingly, nuclear receptors as well as transcription factors implicated in the regulation of energy metabolism are differentially affected by the action of RIP140. The transcriptional activity of PPARα, ATF2 and CREB, in the context of UCP1 promoter, is inhibited in a dose-dependent manner by RIP140 confirming that the balance between RIP140 and PGC-1α relies on the direct antagonism between the two coregulators. Regarding PPARβ/δ, its activity is more sensitive to the action of RIP140 as far less RIP140 is necessary to block the upregulation induced by PGC-1α. Thus, a low level of RIP140 is capable of blocking the action of PGC-1α, depending on the transcriptional context, and it adds a supplementary level of complexity to the balance between RIP140 and PGC-1α coregulator activity. In addition, we cannot exclude that PGC-1α activity is indirectly modulated by RIP140 through AMPK as shown previously [43]. Expression of PGC-1α itself is not affected by modulation of RIP140 protein level but we found that expression of PGC-1β is upregulated in RIP140-null EDL. This is consistent with the ability of PGC-1β to drive the expression of MyHCIIx fibers [44] and might contribute to the modulation of contractile properties observed in RIP140-null mice [25].

Ablation of RIP140 expression leads to a mild fiber type switch in favor of MyHCIIx and MyHCIIa [25]. Interestingly, data from immunohistochemistry experiments show that depletion of RIP140 leads to specific expression of UCP1 in MyHCIIa fibers ruling out the contamination of muscle samples with brown adipocytes. In addition, the difference in UCP1 mRNAs between the RIP140-null EDL and soleus is consistent with the relative proportion of their MyHCIIa myofiber content, 20% and 53% respectively [25] and this increase in oxidative fibers is consistent with improvement of insulin sensitivity and metabolic parameters in RIP140-null mice. UCP1 has been shown to be almost exclusively expressed in the brown fat conferring to this tissue its specific function in non-shivering thermogenesis [37], [45]. Although controversial, there is growing evidence that UCP1 is expressed in other tissues such as the thymus [46], the white adipose tissue and skeletal muscles [21], [23], [47], [48]. Mice devoid of RIP140 have increased oxygen consumption and respiratory exchange ratio compared to WT littermates [25] suggesting they utilize fatty acids as the main source of energy. This is confirmed by elevated β-oxidation rate found in both adipocytes [21] and the EDL of RIP140-null mice (herein). In particular, we have shown that RIP140 acts simultaneously on fatty acid transport and mitochondria number and activity [25], [35] to ultimately alter β-oxidation rate in skeletal and cardiac muscles. Absence of RIP140 leads to ectopic expression of UCP1 in the white adipose tissue [23] suggesting that mitochondrial uncoupling might be implicated as well. The induction of UCP1 and the conversion of white adipocytes to a brown fat phenotype have also been achieved by the ectopic expression of PGC-1α [32]. Recently, we have shown that RIP140 regulates UCP1 expression through histone and DNA methylation in adipocytes [36]. However, our bisulfite sequencing experiments failed to show any changes in the methylation status of UCP1 promoter in RIP140-null soleus compared to WT. Surprisingly, we found that UCP1 promoter is hypomethylated suggesting that the local chromatin displays a permissive state compatible with active regulation of UCP1 expression in skeletal muscles. This is consistent with our data demonstrating that PGC-1α, PPARα, PPARβ/δ and ERRα are capable of regulating UCP1 promoter activity in muscle cells in the same way as in adipocytes. In addition, expression of UCP1 and its potential regulation by cold through activation of ATF2 and CREB transcription factors, in skeletal muscle is relevant in the light of recent data suggesting that skeletal muscle shares the same lineage as brown adipose tissue [49], [50].

The control of glucose uptake into skeletal muscle by regulation of the expression and trafficking of specific transporters is essential for the maintenance of euglycaemia. In this study, we show that absence of RIP140 leads to increased GLUT4 trafficking and glucose uptake in the soleus independently of insulin. Although GLUT4 mediates insulin-dependent glucose transport, this is consistent with data from GLUT4-muscle specific knockout mice showing that GLUT4 participates in the basal glucose uptake in the EDL [51]. The mechanism by which GLUT4 trafficking is increased relies on activation of AMPK due to ectopic expression of UCP1 and increased, although not significant, energy dissipation. These data are consistent with previous studies demonstrating that ectopic expression of UCP1 in skeletal muscle leads to upregulation of AMPK activity and increased glucose uptake preventing diet-induced obesity and insulin resistance [52], [53]. Metformin, a drug widely used to treat insulin resistance in obese and type 2 diabetic patients, improves glucose disposal by skeletal muscle and affects hepatic glucose production [54]. Metformin is thought to act mainly by enhancing AMPK activity through changes in the AMP to ATP ratio [19], [20]. AMPK plays an important role in the beneficial effects of metformin and is part of the mechanism by which contraction-induced glucose uptake is achieved. In adipocytes, it was recently demonstrated that cytoplasmic RIP140 modulates GLUT4 trafficking through a mechanism that is distinct from transcriptional regulation [55]. This is the consequence of RIP140 interacting with AS160 and impeding its inactivation by Akt. High fat diet stimulates RIP140 nuclear export in white adipose tissue and blocks transcriptional regulating properties of RIP140 [56]. In skeletal muscle, RIP140 is preferentially localized in the nucleus (data not shown) but we cannot rule out the possibility that RIP140 may also be localized in the cytoplasm or that high fat diet affects its localization. This provides an additional mechanism that might be implicated in the regulation of glucose uptake in the EDL and should be investigated further.

In RIP140 transgenic mice, AMPK activity is inhibited in the soleus. This is accompanied by decreased β-oxidation that reflects alteration in expression of genes implicated in fatty acid binding and transport, with no change in the uptake of glucose. This suggests that there is a minimal threshold of glucose uptake that is maintained in the soleus and, similar to the state of β-oxidation in the EDL, it is refractory to further inhibition by increased RIP140 and subsequent inhibition of AMPK phosphorylation/activation. Because UCP1 is normally undetectable in skeletal muscles, the effect of exogenous RIP140 on AMPK cannot be explained by alteration in mitochondrial uncoupling. AMPK exists in the cell as heterotrimeric complexes with different combinations of isoforms displaying selective affinity for its allosteric activator, AMP [57]–[59]. Interestingly, we detect increased expression of subunits α (herein) and γ3 [25] in RIP140 transgenic soleus suggesting that the decrease in AMPK activity could be due to the formation of specific complexes with lower activity. In addition, RIP140 seems to act as a transcriptional coactivator for these genes as found in a number of other cell systems [42], [60], [61] and this should be investigated further.

In conclusion, this study shows that depletion of RIP140 improves the metabolic parameters of both glycolytic and oxidative muscles which participates in the overall phenotype of RIP140-null mice and suggest that RIP140 might be a potential therapeutic target in the treatment of insulin resistance in obese and type 2 diabetic patients.

## Methods

### Ethics statement

All animal studies were carried out according to UK Home Office guidelines.

### Animals

The generation of RIP140-null and RIP140 transgenic mice has been described previously [22], [35].

### Expression analysis

The expression of target genes was determined using SYBR green reagent and gene-specific primers on the opticon 2 PCR machine (MJ Research). Expression levels were normalized to ribosomal coding gene RPL7.

### Western-blot analysis

Muscle tissues were homogenized in buffer containing 50 mM HEPES (pH 7.6), 150 mM NaCl, 5 mM EDTA, 1% NP40 with a cocktail of phosphatase and protease inhibitors (SIGMA). Protein concentration was determined by using the Bradford method (Bio-Rad). Samples were resuspended in Laemmli buffer and proteins separated on SDS-PAGE. Proteins were transferred to PVDF membranes (GE healthcare) and subjected to western blot analysis. After incubation with primary antibodies, membranes were incubated with secondary antibodies linked to horseradish peroxidase (Dako). Quantification was made using NIH Image J software. Primary antibodies used in this study were from Santa-Cruz against PPARα (sc-9000), FABP3 (sc-58274) and GLUT4 (sc-7938); from Cell signaling against Akt (9272), phospho-Akt (9276), AMPK (2532), phospho-AMPK (2531), ACC (3662), phospho-ACC (3661); from SIGMA against UCP1 (U6382) and PPARβ/δ (SAB2500812); from Novus Biologicals against ERRα (NLS5402); from Abcam against actin (ab8226) and mouse monoclonal anti-RIP140 (6D7) was made in house.

### β-oxidation

Isolated muscles were pre-incubated for 30 min in Krebs-Henseleit bicarbonate buffer (KRH: 25 mM NaHCO_3_, 118 mM NaCl, 4.7 mM KCl, 1.2 mM MgSO_4_, 1.2 mM NaH_2_PO_4_ and 1.2 mM CaCl_2_) with 20 mg/ml free-fatty acid BSA, 5 mM glucose. The muscles were then transferred to KRH buffer containing 20 mg/ml free-fatty acid BSA+0.3 mM L-Carnitine and 12 µM ^3^[H]-palmitic acid (3 µCi/well) and incubated for 2 hours. The media was transferred to tubes containing one tenth volume of TCA 50%. After centrifugation, the supernatant was transferred to fresh tubes containing half volume of chloroform∶methanol (2∶1). After centrifugation, the aqueous phase was then transferred to scintillation vials and the ^3^H_2_O production was then measured with a scintillation counter. Then, muscles were incubated with 0.1% SDS for 2 hours at 50°C and the extract was assayed for protein concentration to normalize.

### Glucose uptake

Isolated muscles were glucose starved by pre-incubating for 2 hours in KRH buffer. The muscles were then transferred to KRH buffer containing 20 mg/ml free-fatty acid BSA+^3^H-2-deoxyglucose (2 µCi/well) with 5 mM non-radioactive 2-deoxyglucose for 5 min. The muscles were rapidly washed 3 times with ice-cold KRH buffer to stop the reaction. Then the muscles were transferred to eppendorf tubes with 0.1% SDS and incubated for 2 hours at 50°C. The extract was counted for radioactivity and assayed for protein concentration to normalize.

### Immunohistochemistry

Immunostaining was performed essentially as described previously [25] using primary antibodies from Santa-Cruz against GLUT4 (sc-7938), UCP1 (sc-6528) and mouse monoclonals against MyHCIIa (SC-71) and MyHCIb (BAF8). Monoclonals for MyHC were a gift from Dr A. Rowlerson.

### Promoter assays

C2C12 cells (purchased from ATCC) were transfected by using lipofectamine 2000 (Invitrogen) with UCP1 (4 kb)-luciferase reporter [21] together with vectors expressing RXR, PPARα, PPARβ/δ, CREB (gift of Dr D.M. Stocco), ERRα, ATF2 (gift of Dr M. Sabbah), RIP140 and PGC-1α as indicated. After overnight incubation, cells were treated with 100 µM WY14643, 10 µM GW501516, 10 µM XCT790, 10 µM Forskolin (Calbiochem) or vehicle as indicated and harvested 24 h later for the determination of luciferase and GFP (used to correct for differences in transfection efficiencies) activities.

### Preparation of skinned myofibers

Muscles isolated from mice were incubated in cooled relaxing solution A containing 2.77 mM CaK_2_EGTA, 7.23 mM K_2_EGTA, 6.56 mM MgCl_2_, 0.5 mM DTT, 50 mM K-MES, 20 mM Imidazole, 20 mM Taurine, 5.3 mM Na_2_ATP, 15 mM Phosphocreatine (pH 7.1). The myofibers were separated out and transferred into cooled solution A containing 50 µg/mL of saponin. After, incubation at mild stirring for 30 min for complete solubilisation of the sarcolemma, the skinned myofibers were washed three times 10 min in solution B containing 2.77 mM CaK_2_EGTA, 7.23 mM K_2_EGTA, 1.38 mM MgCl_2_, 0.5 mM DTT, 100 mM K-MES, 20 mM Imidazole, 20 mM Taurine, 3 mM K_2_HPO_4_ (pH 7.1).

### Mitochondrial oxygen consumption

Respiration of skinned myofibers was measured using a Clark type electrode sensitive to oxygen in solution B containing respiratory substrates and 2 mg/ml BSA. Skinned myofibers from WT and RIP140-null soleus were energized with 5 mM pyruvate/2 mM malate then 135 µM of palmitate was added to activate uncoupling. UCP1-dependent uncoupling was determined in the presence of 5 mM CAT and 0.5 mM GDP. Then 5 mM FCCP was added to the chamber to test maximal capacity of mitochondria preparation.

### Statistical analysis

All data were analyzed by Student's t-test. Significance level was preset to p<0.5. Data are presented as mean ± SEM across experiments.

## Supporting Information

Figure S1 **Expression of GLUT4 is decreased in the RIP140 transgenic EDL.** Analysis of GLUT4 and tubulin expression in RIP140 transgenic (TG) and WT EDL by western blot.
(TIF)

Figure S2 **RIP140 does not alter Akt activity.** (A) Analysis of phospho-Akt and total Akt expression on RIP140-null (KO), transgenic (TG) and WT soleus (SOL) and EDL by western-blot. (B) Quantification of phospho-Akt to Akt ratio for western-blot expressed as mean ± SEM.
(TIF)

Figure S3 **RIP140 does not alter AMPK activity in the EDL.** Analysis of phospho- αAMPK, total α and β AMPK expression on RIP140-null (KO), transgenic (TG) and WT EDL by western-blot. Quantification of phospho-AMPK to AMPK ratio for western-blot expressed as mean ± SEM.
(TIF)

Figure S4 **Specificity of UCP1 antibody tested for immunochemistry.** Immunohistochemistry for UCP1 on WT, RIP140-null (KO) and UCP1-null (KO) soleus. Sections incubated with primary antibody omitted are displayed as negative control (Neg).
(TIF)

Figure S5 **Depletion of RIP140 does not alter the expression of other mitochondrial uncoupling proteins in the soleus.** Real-time RT-PCR analysis of Ant1, Ant2, UCP2, and UCP3 in the soleus (SOL) of RIP140-null (KO) and WT mice. Data are expressed as mean ± SEM.
(TIF)

Figure S6 **UCP1 proximal promoter is hypomethylated in the soleus.** Bisulfite sequencing experiments were performed on muscles isolated from the soleus (SOL) of RIP140-null (KO) and WT mice. (n = 12–15).
(TIF)

Figure S7 **UCP1 promoter activity is inducible in C2C12.** Cells were transfected with UCP1(4 kb)-luciferase reporter and vectors expressing (A) PPARα, (B) PPARβ/δ, (C) ERRα, (D) CREB or ATF2, and treated with (A) 100 µM WY14643 (WY), (B) 10 µM GW501516 (GW), (C) 10 µM XCT790 (XC), (D) 10 µM Forskolin (Fo) or vehicle (-) as indicated. Data are expressed as mean ± SEM, *p<0.05 *vs* vehicle.
(TIF)
