# Supplementary figures and images for: Absence of RIP140 Reveals a Pathway Regulating glut4-Dependent Glucose Uptake in Oxidative Skeletal Muscle through UCP1-Mediated Activation of AMPK
Source: PLoS One. 2012 Feb 28;7(2):e32520. doi: 10.1371/journal.pone.0032520 (PMC3289711; doi:10.1371/journal.pone.0032520)

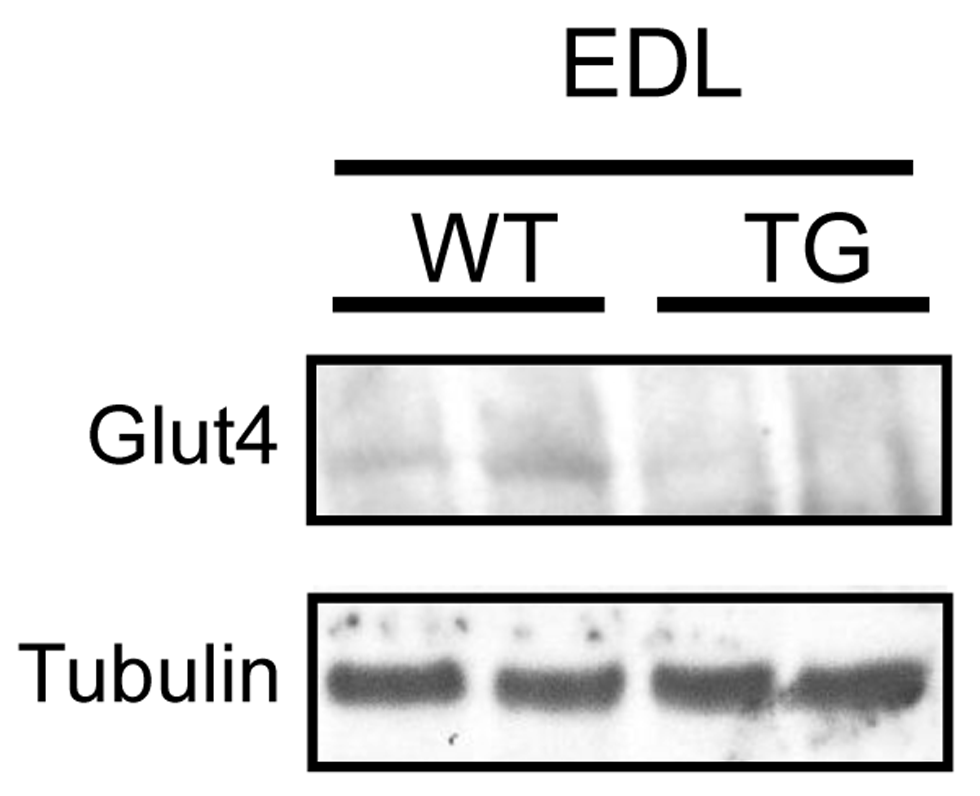

Supplement: Figure S1 — Expression of GLUT4 is decreased in the RIP140 transgenic EDL. Analysis of GLUT4 and tubulin expression in RIP140 transgenic (TG) and WT EDL by western blot. (TIF) [file pone.0032520.s001.tif]

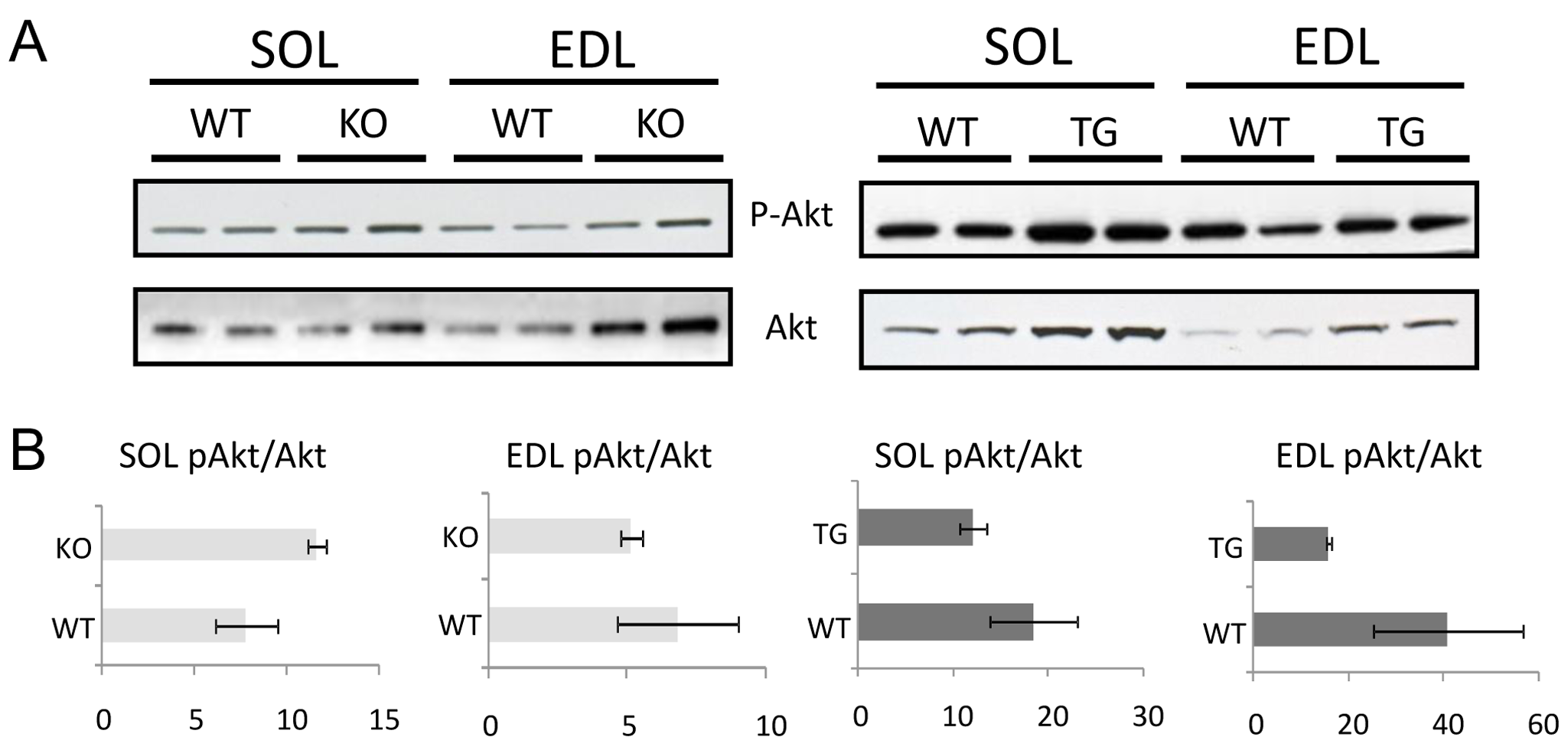

Supplement: Figure S2 — RIP140 does not alter Akt activity. (A) Analysis of phospho-Akt and total Akt expression on RIP140-null (KO), transgenic (TG) and WT soleus (SOL) and EDL by western-blot. (B) Quantification of phospho-Akt to Akt ratio for western-blot expressed as mean ± SEM. (TIF) [file pone.0032520.s002.tif]

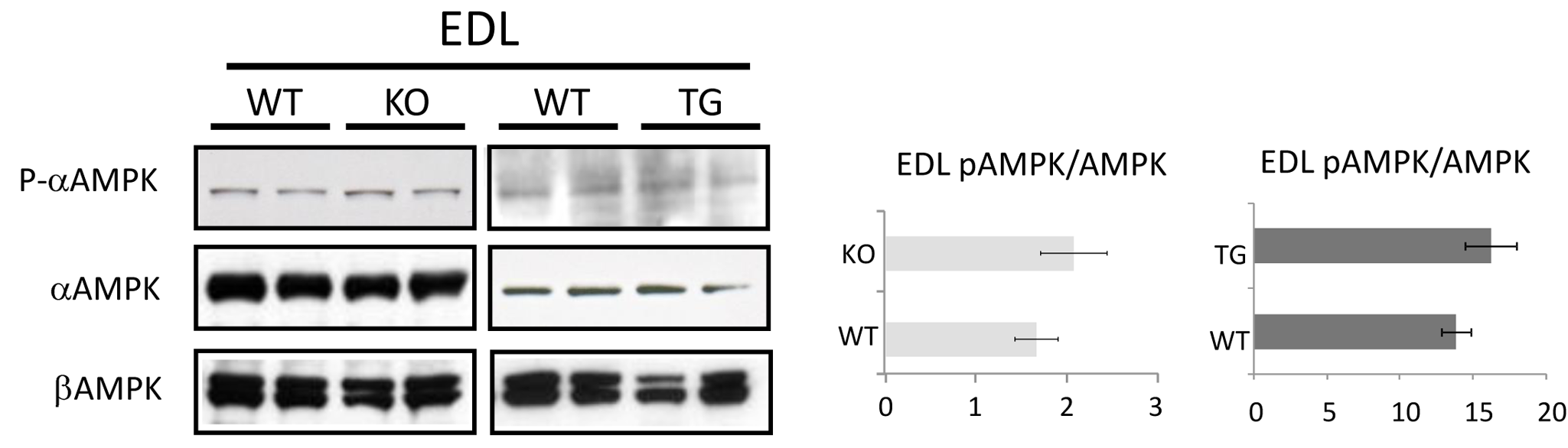

Supplement: Figure S3 — RIP140 does not alter AMPK activity in the EDL. Analysis of phospho- αAMPK, total α and β AMPK expression on RIP140-null (KO), transgenic (TG) and WT EDL by western-blot. Quantification of phospho-AMPK to AMPK ratio for western-blot expressed as mean ± SEM. (TIF) [file pone.0032520.s003.tif]

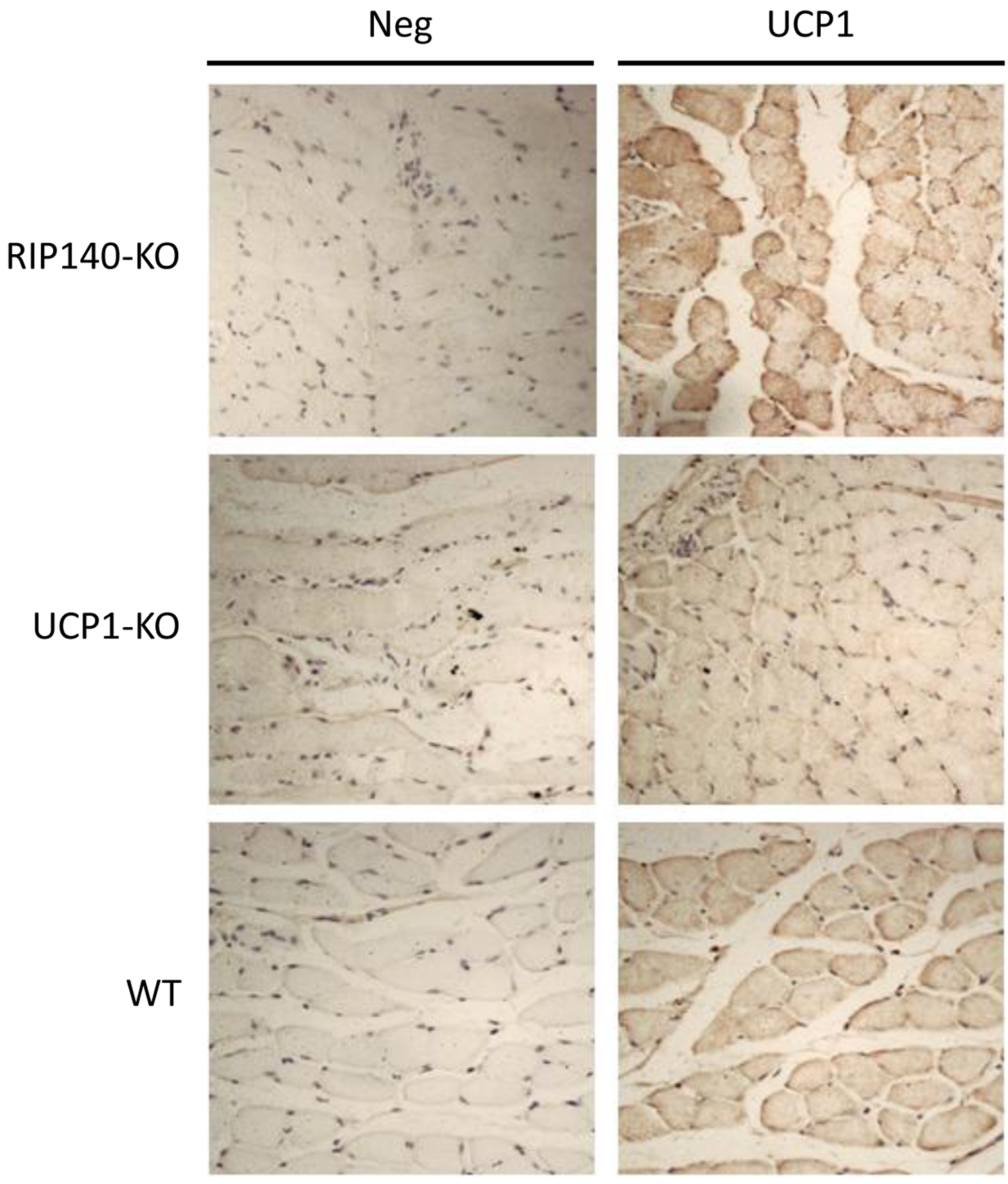

Supplement: Figure S4 — Specificity of UCP1 antibody tested for immunochemistry. Immunohistochemistry for UCP1 on WT, RIP140-null (KO) and UCP1-null (KO) soleus. Sections incubated with primary antibody omitted are displayed as negative control (Neg). (TIF) [file pone.0032520.s004.tif]

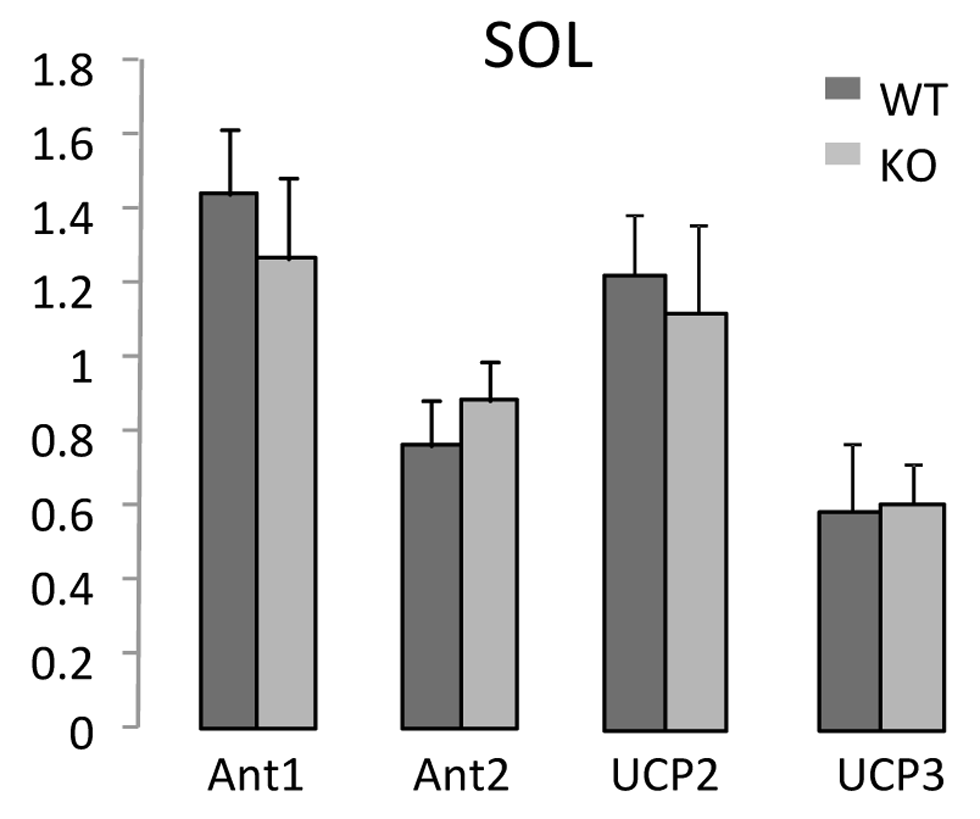

Supplement: Figure S5 — Depletion of RIP140 does not alter the expression of other mitochondrial uncoupling proteins in the soleus. Real-time RT-PCR analysis of Ant1, Ant2, UCP2, and UCP3 in the soleus (SOL) of RIP140-null (KO) and WT mice. Data are expressed as mean ± SEM. (TIF) [file pone.0032520.s005.tif]

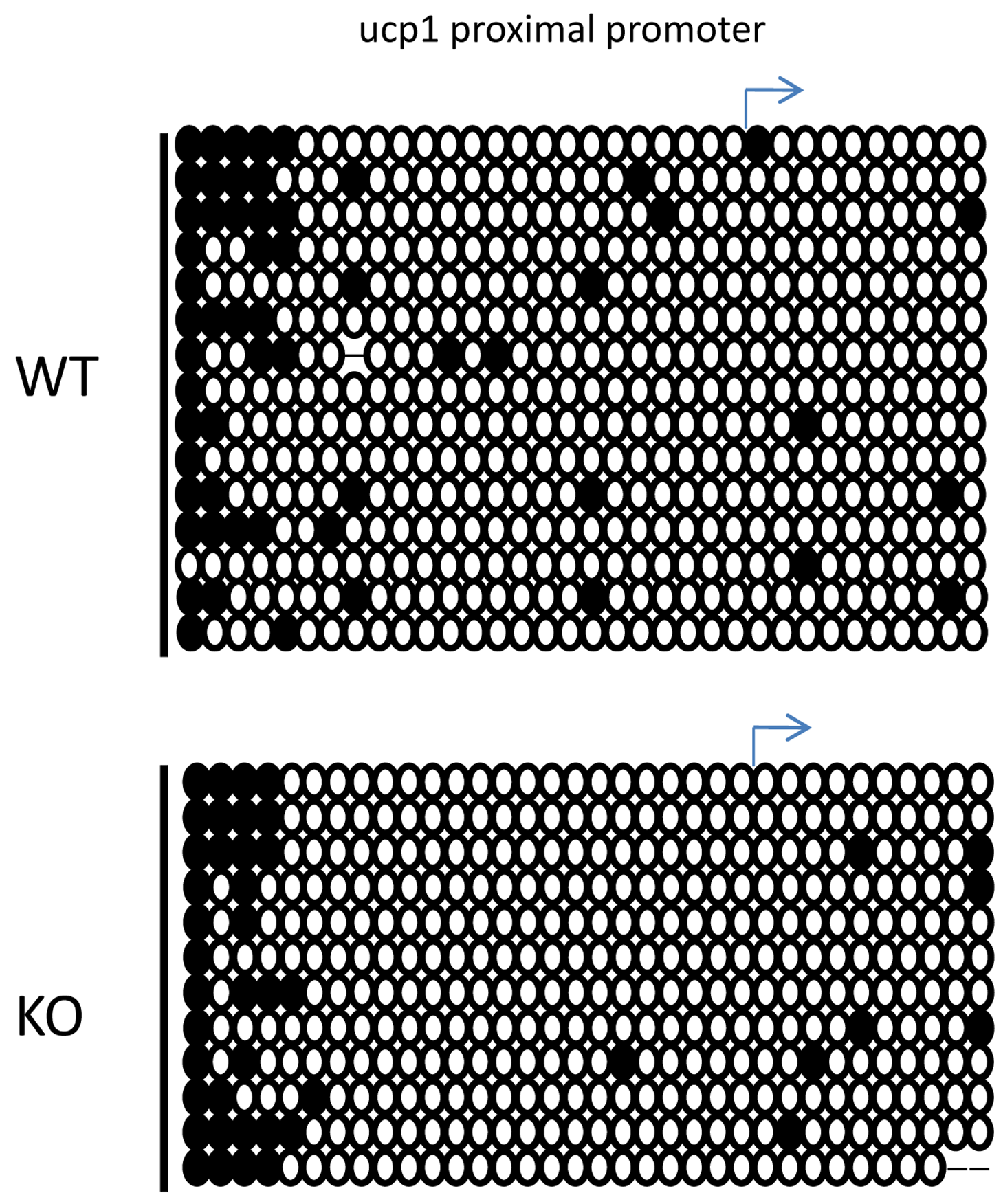

Supplement: Figure S6 — UCP1 proximal promoter is hypomethylated in the soleus. Bisulfite sequencing experiments were performed on muscles isolated from the soleus (SOL) of RIP140-null (KO) and WT mice. (n = 12–15). (TIF) [file pone.0032520.s006.tif]

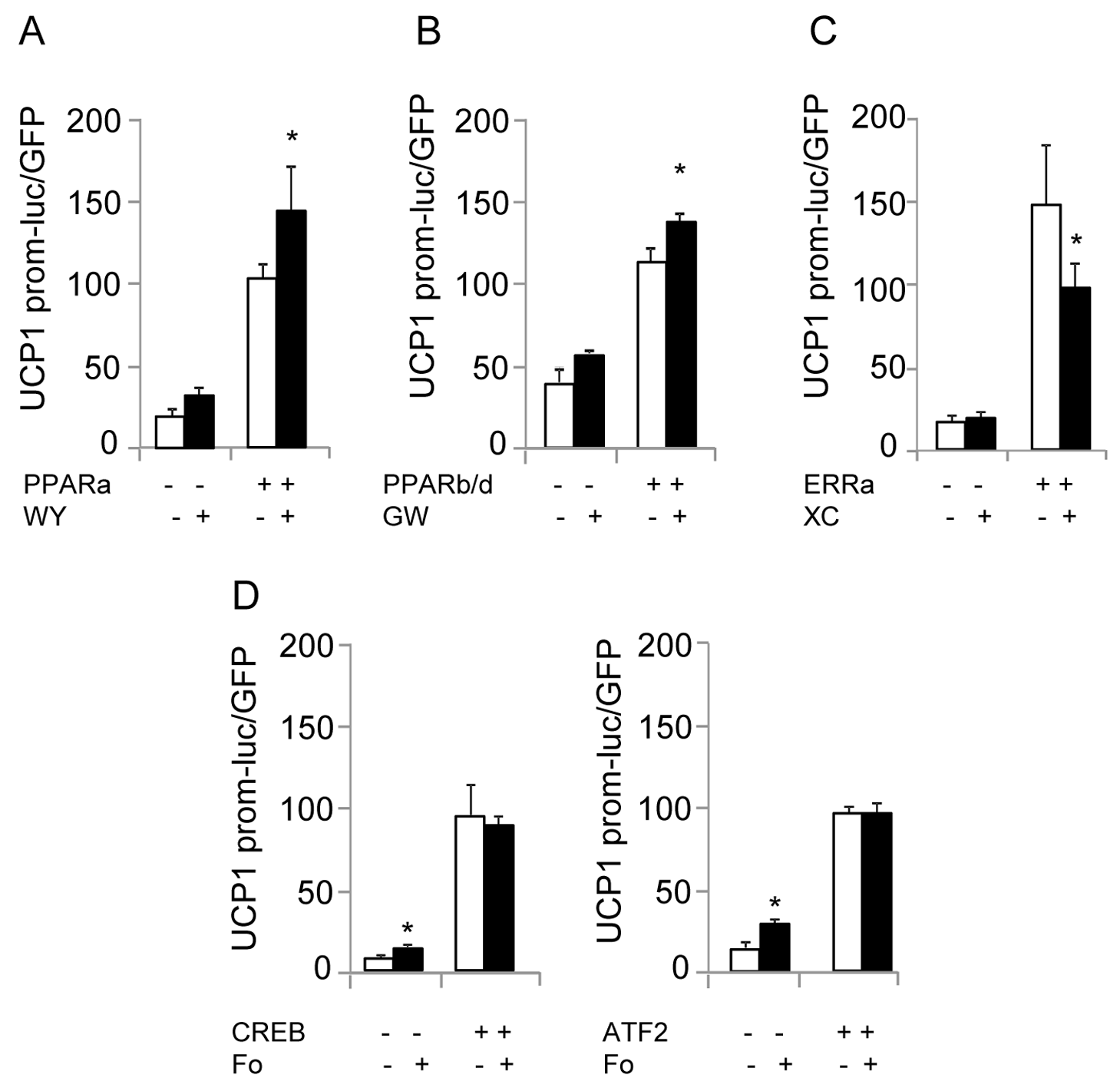

Supplement: Figure S7 — UCP1 promoter activity is inducible in C2C12. Cells were transfected with UCP1(4 kb)-luciferase reporter and vectors expressing (A) PPARα, (B) PPARβ/δ, (C) ERRα, (D) CREB or ATF2, and treated with (A) 100 µM WY14643 (WY), (B) 10 µM GW501516 (GW), (C) 10 µM XCT790 (XC), (D) 10 µM Forskolin (Fo) or vehicle (-) as indicated. Data are expressed as mean ± SEM, *p<0.05 vs vehicle. (TIF) [file pone.0032520.s007.tif]
